# Memory Beliefs Drive the Memory Bias on Value-based Decisions

**DOI:** 10.1038/s41598-018-28728-9

**Published:** 2018-07-12

**Authors:** Tehilla Mechera-Ostrovsky, Sebastian Gluth

**Affiliations:** Department of Psychology, University of Basel, Missionsstrasse 62a, 4055 Basel, Switzerland

## Abstract

For many value-based decisions, people need to retrieve relevant information from their memory. In our previous work, we have shown that memory biases decisions in the sense that better-memorized choice options are preferred, even if these options are comparatively unattractive. However, the cognitive mechanisms that drive this memory bias remain unclear. In the current pre-registered study, we tested the hypothesis that the memory bias arises because people believe they remember better options more often than worse options. Specifically, we predicted a positive correlation between the memory bias on value-based decisions and the belief in value-dependent memory performance. This prediction was confirmed. Additional exploratory analyses revealed that memory performance was indeed higher for more attractive options, indicating that letting decisions be influenced by memory can be an adaptive strategy. However, the memory bias persisted after correcting for this effect, suggesting that it is not simply an artifact of unequal memory performance. Our results highlight a critical influence of beliefs on behavior and add to an emerging understanding of the role of memory in shaping value-based decisions.

## Introduction

Many, if not all, value-based decisions necessitate sampling relevant information from memory^1–6^, in particular when options are not within reach. For example, consider yourself brooding over potential resorts for your next skiing holidays. To make this decision, you may retrieve available options from your memory, such as the last three resorts you have been to in recent years. Let us further assume that you can only recall two of the three resorts, and your partner has to remind you of the forgotten option. How likely is it that you will prefer this forgotten option over the remembered ones? Perhaps the mere fact that you were not able to remember this skiing resort should be taken as evidence against it. Thus, episodic memories and beliefs about these memories may influence value-based decisions in every-day life.

In our previous work, we investigated the cognitive and neural mechanisms of value-based decisions from memory^2^. Thereto, participants repeatedly chose between two out of a set of food snacks which were not presented directly but had to be retrieved from memory. We found that the ability to remember an option had a biasing influence on decisions: If one of the two options was forgotten, participants preferred the other (remembered) option–even if this option’s subjective value was below average (i.e., even if choosing the forgotten option would have been optimal). On the neural level, the influence of this memory bias on decisions could be linked to increased effective connectivity between the hippocampus and the ventromedial prefrontal cortex, two brain regions known to play fundamental roles in both memory and decision-making processes^6–8^. Yet, the study did not address the question of *why* people exhibit a memory bias. In other words, what are the cognitive mechanisms that drive the memory bias in value-based decisions from memory?

**Figure 1:**
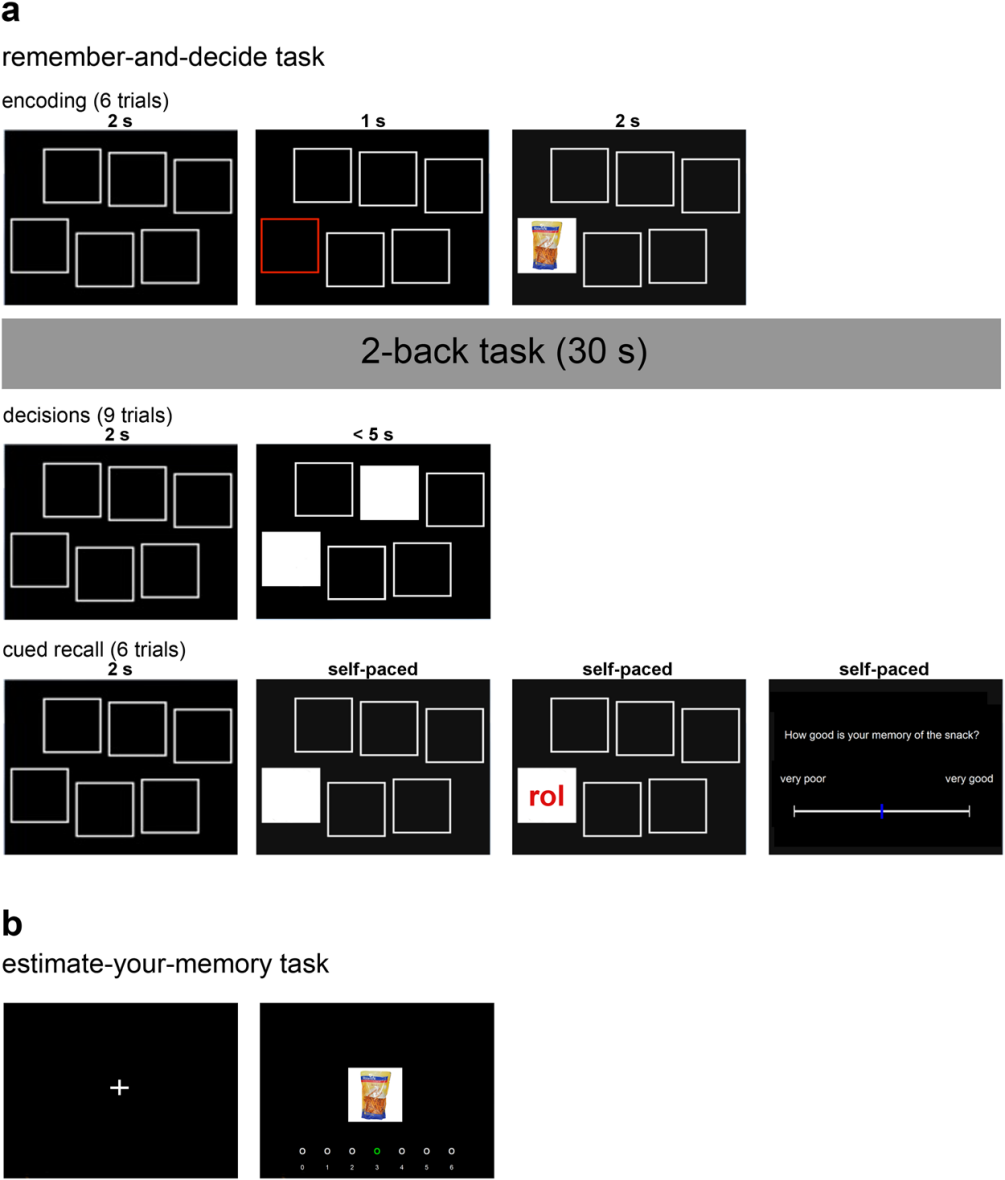
Core tasks of the study. (**a**) The remember-and-decide task consisted of 24 blocks, each comprising multiple sub-tasks (depicted are single trials for each sub-task of a single block): first, participants encoded six snack-location associations; after a 2-back task (used to overwrite working memory), they repeatedly decided between pairs of snacks whose identity had to be retrieved from memory; finally, they had to report the identity of each snack via cued recall and how well they remembered the snack on a continuous rating scale. (**b**) In the estimate-your-memory task, participants were asked to estimate how often (out of 6 times) they had recalled each snack during the remember-and-decide task.

In the current study, we tested the hypothesis that the influence of memory on decision making is mediated by beliefs about memory. More specifically, we predicted that people believe to remember more valuable choice options with higher accuracy than less valuable options. As a consequence, they discount forgotten options for the mere fact that they are forgotten (as in the skiing resort example above), thus exhibiting a memory bias. Following this reasoning, we predicted a positive correlation between the degree to which people estimate their memory to be better for more valuable options and the strength of the memory bias. To test these hypotheses, we asked 90 participants to complete the *remember-and-decide* task from our previous work with minor modifications (Fig. 1a and Methods), followed by a novel *estimate-your-memory* task (Fig. 1b). In this second task, participants estimated for every snack how often they have remembered the snack during the remember-and-decide task (importantly, all snacks were shown equally often during the remember-and-decide task; see Methods).

## Results

### Hypothesis-driven Analyses

We pre-registered our study design on the Open Science Framework (OSF) website (https://osf.io/ze8xh/) with the following three hypotheses: H1) As in our previous work, participants exhibit a memory bias on decisions in the remember-and-decide task. H2) Participants report higher memory retrieval success for more valuable options in the estimate-your-memory task. H3). The strength of the memory bias and the value-dependency of memory estimation are positively correlated across participants.

**Figure 2:**
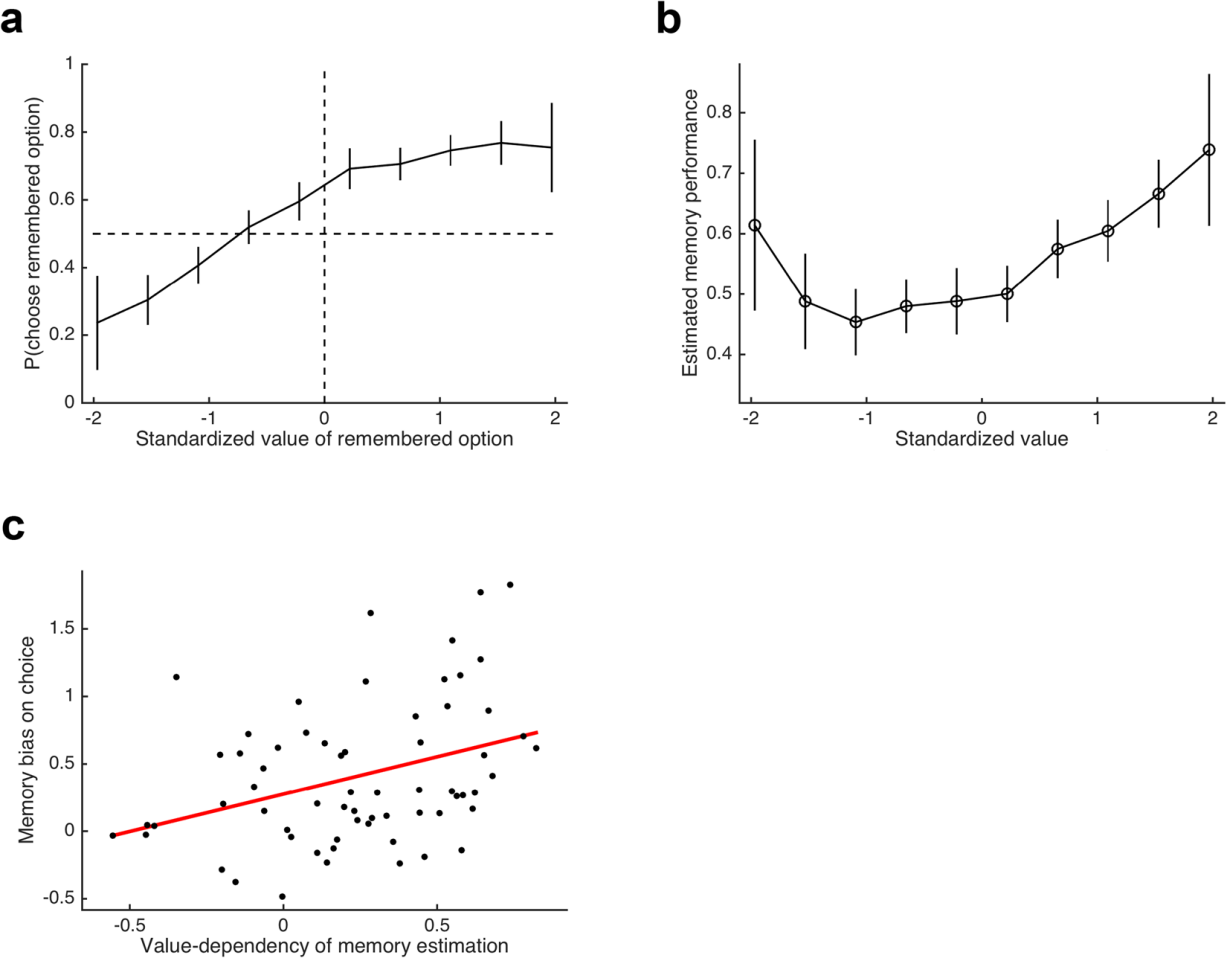
Results of hypothesis-driven analyses. (**a**) Probability of choosing a remembered option (over a forgotten option) as a function of its standardized value; the dashed vertical and horizontal lines help to illustrate the presence of a memory bias. (**b**) Estimation of successful memory retrieval as a function of value; participants reported to have remembered more valuable options more frequently. (**c**) The value-dependent memory estimation and the memory bias were positively correlated. Error bars in all figures represent 95% CIs.

To test for the memory bias (H1), we followed our previous work and conducted a logistic regression in each participant for those decision trials in the remember-and-decide task, in which one option was remembered and the other was forgotten (according to the cued recall phase of the remember-and-decide task; see Fig. 1a). The decision for/against the remembered option was regressed against the standardized value assigned to this option. A rational (i.e., unbiased) agent would be indifferent between a forgotten option and an average remembered option (i.e., an option with a standardized value of 0). A deviation from this behavior in line with the memory bias can be illustrated by a shift of the regression curve to the left and quantified by a regression intercept b_0,H1_ greater than 0. Indeed, the average estimated b_0,H1_ was significantly positive (*t*_63_ = 6.23, *P < 0.001*, Cohen’s *d* = 0.78; Fig. 2a). Thus, we could replicate the memory bias in the current study, meaning that when one option was forgotten, participants’ preference was shifted towards the remembered option even if this option was comparatively unattractive.

Next, we tested whether the estimation of successful memory retrieval of an option depends on the option’s value (H2). Thereto, we conducted a linear regression analysis in each participant with the options’ value and estimated memory performance serving as independent and dependent variables, respectively. The regression slope b_1,H2_ indicates whether memory estimation depends on value, with a positive slope indicating that better options were estimated to be remembered better. As predicted, the average estimated b_1,H2_ was significantly positive (*t*_63_ = 5.67, *P* < 0.001, *d* = 0.71; Fig. 2b). In other words, participants estimated their memory to be better for desired options than for undesired options.

Our final and most critical hypothesis (H3) was that the memory bias and value-dependency of memory estimation are positively linked to each other. To test this prediction, we performed a Pearson product-moment correlation between the two regression coefficients b_0,H1_ and b_1,H2_ from the previous analyses. In line with our hypothesis, this analysis revealed a significantly positive correlation (*r*_62_ = 0.36, *P* = 0.002), suggesting that individuals with a stronger belief in value-dependent memory performance exhibited a stronger memory bias on value-based decisions. To summarize, the results of our hypothesis-driven analyses confirm the existence of a memory bias in value-based decisions from memory and furnish the belief in value-dependent memory abilities as an explanation to its origin.

### Exploratory Analyses

In addition to our hypothesis-driven analyses, we also conducted a series of exploratory data analyses (inferential statistics for these analyses are provided for conventional reasons). First of all, we assessed to what extent the results of the hypothesis-driven tests depended on the comparatively high rate of excluded participants (we excluded 26 participants for the above-mentioned analyses based on several pre-registered exclusion criteria; see Supplementary Information). When testing our hypotheses with the full sample of 90 participants, we obtained equivalent results (Table 1). Some effects were even stronger (e.g., the correlation between belief and bias increased to 0.45). For the following exploratory analyses, we report results of the restricted sample, but again, results of the full sample did not differ qualitatively (see Table 1 and Supplementary Information).

**Table 1:** Summary of statistical results for restricted and full samples.

	Restricted sample (*n* = 64)	Full sample (*n* = 90)
Hypothesis-driven analyses		
H1: Memory bias	*t*_63_ = 6.23*** d = 0.78	*t*_89_ = 6.44*** d = 0.68
H2: Value-dependent memory estimation	*t*_63_ = 5.67*** d = 0.71	*t*_89_ = 6.90*** d = 0.73
H3: Correlation between memory bias and value-dependent memory estimation	*r*_62_ = 0.36**	*r*_88_ = 0.45***
Exploratory analyses		
Quadratic effect of value on memory estimation	*t*_63_ = 5.90*** d = 0.74	*t*_89_ = 5.66*** d = 0.60
Linear effect of value on memory performance	*t*_63_ = 4.56*** d = 0.57	*t*_89_ = 4.72*** d = 0.50
Quadratic effect of value on memory performance	*t*_63_ = 3.85*** d = 0.48	*t*_89_ = 4.23*** d = 0.45
Model comparison: linear-and-quadratic > linear-only on memory estimation	*χ*^2^_63_ = 102.3**	*χ*^2^_89_ = 134.7**
Model comparison: linear-and-quadratic > quadratic-only on memory estimation	*χ*^2^_63_ = 220.9***	*χ*^2^_89_ = 258.1***
Model comparison: linear-and-quadratic > linear-only on memory performance	*χ*^2^_63_ = 157.2***	*χ*^2^_89_ = 245.7***
Model comparison: linear-and-quadratic > quadratic-only on memory performance	*χ*^2^_63_ = 321.0***	*χ*^2^_89_ = 400.1***
Linear effect of memory performance on memory estimation	*t*_63_ = 14.18*** d = 1.77	*t*_89_ = 18.24*** d = 1.92
Linear effect of value on memory estimation when controlling for memory performance	*t*_63_ = 4.77*** d = 0.60	*t*_89_ = 5.43*** d = 0.57
Standardized value of forgotten options	*t*_63_ = −4.98*** d = −0.62	*t*_89_ = −6.10*** d = −0.64
Corrected memory bias	*t*_63_ = 5.84*** d = 0.73	*t*_89_ = 5.65*** d = 0.60

Note: Results of the path analyses for the full sample are provided in the Supplementary Information. **P < 0.01, ***P < 0.001.

Next, we tested whether there was a quadratic relationship between value and memory estimation beyond the above mentioned linear effect. The seemingly U-shaped pattern of memory estimation as shown in Fig. 2b suggests that participants estimated their memory for the worst options to be higher than for medium-to-low options. To test this more systematically, we added a quadratic term of the (standardized) value to the regression analysis of H2. Indeed, this quadratic term was positive across participants (consistent with a U-shaped relationship), and would be significant when using a statistical test (*t*_63_ = 5.90, *P* < 0.001, *d* = 0.74). Importantly, adding the quadratic term did not diminish the contribution of the linear term. To provide further evidence for the presence of both linear and quadratic effects of value on memory estimation, we compared the linear-only, the quadratic-only, and the linear-and-quadratic regression models via likelihood ratio tests^9^. The increased complexity of the linear-and-quadratic model was clearly justified by the substantial improvement in model fit (comparison with linear-only: *χ*^2^_63_ = 102.3, *P* = 0.001; comparison with quadratic-only: *χ*^2^_63_ = 220.9, *P* < 0.001). Thus, participants’ value-dependency of memory estimation is best characterized by assuming both a linear and a quadratic component such that not the worst but the medium-to-low value options are assumed to be forgotten most often.

**Figure 3:**
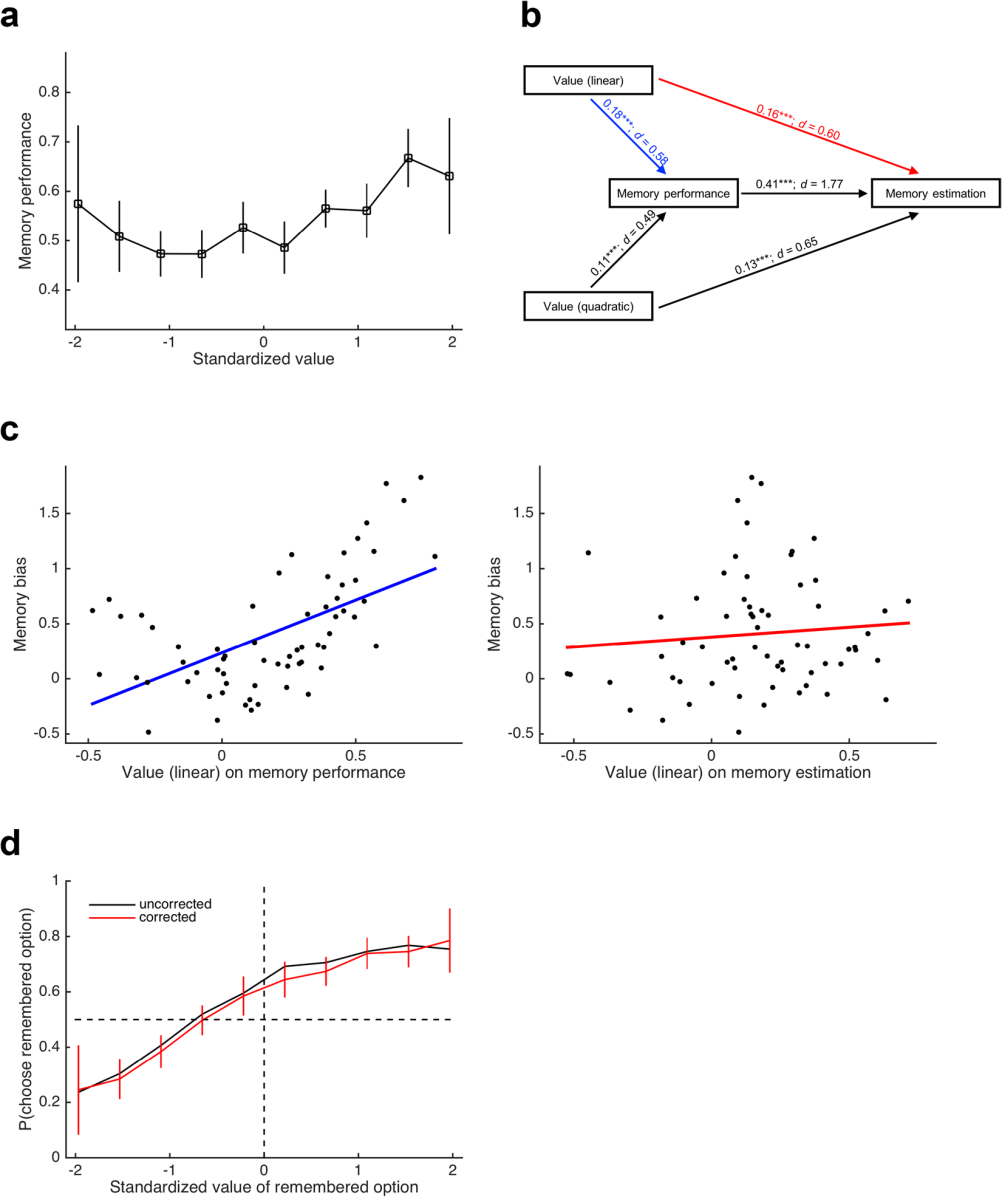
Results of exploratory analyses. (**a**) Actual memory performance as a function of value; similar to memory estimation, memory performance was influenced by linear and quadratic value terms. (**b**) Path analysis of the interplay of value, memory performance, and memory estimation; the path analysis was conducted in each participant, and path coefficients were tested against 0 at the group level (all coefficients would be significant at ***P < 0.001); the influences of value on memory estimation were partially mediated by performance, but there were also direct influences. (**c**) Correlation between the memory bias and the path coefficients from Value (linear) on Memory performance (left panel; blue path in (**b**)) and on Memory estimation (right panel; red path in (**b**)); the memory bias was correlated with the “true” dependency of memory performance on value but not with participants’ overestimation of this dependency. (**d**) Memory bias before (black) and after (red) correction for the dependency of memory performance on value; for display purposes, CIs are shown for the corrected memory bias only; the memory bias is slightly reduced but still evident after correction.

Similar to memory estimation, we investigated whether actual memory performance depended on value in both a linear and a quadratic way. A logistic regression of memory performance (in the cued recall phase of the remember-and-decide task) on linear and quadratic terms of value indeed provided clear evidence for the presence of both effects (linear: *t*_63_ = 4.56, *P* < 0.001, *d* = 0.57; quadratic: *t*_63_ = 3.85, *P* < 0.001, *d* = 0.48; Fig. 3a), but note that the effect sizes were smaller than those for memory estimation. Model comparisons between the linear-and-quadratic regression model against the linear-only model (*χ*^2^_63_ = 157.2, *P* < 0.001) and against the quadratic-only model (*χ*^2^_63_ = 321.0, *P* < 0.001) support the presence of both effects. Note that these results were surprising, as we did not find evidence for them in our previous study (ref.^2^ see also Discussion).

These results seem to imply that the value-dependency of memory estimation was at least partially driven by participants’ fairly accurate knowledge about how often they had remembered each item in the remember-and-decide task (another indication of this are the high correlations between the linear value-coefficients for memory estimation and performance [*r*_62_ = 0.66, *P* < 0.001] as well as between the quadratic value-coefficients for memory estimation and performance [*r*_62_ = 0.50, *P* < 0.001]). Accordingly, we first ensured that the influence of value on memory estimation was not simply an epiphenomenon of participants’ knowledge that they remembered more valuable items better. Thereto, we added memory performance as an independent variable when regressing memory estimation on (linear and quadratic) value. Memory performance was strongly related to memory estimation (*t*_63_ = 14.18, *P* < 0.001, *d* = 1.77). Importantly, however, adding this variable did not nullify the effect of value on memory estimation (*t*_63_ = 4.77, *P* < 0.001, *d* = 0.60; an additional control analysis to disambiguate between the contributions of value and memory performance to memory estimation is provided in the Supplementary Information). Additionally, we set up a path analysis for each participant to disambiguate the indirect (i.e., performance-mediated) from any direct relationships between value and memory estimation (Fig. 3b). We obtained evidence for a mediation of (linear and quadratic) value on memory estimation by memory performance but also for direct effects of (linear and quadratic) value on memory estimation that could not be explained by participants’ knowledge. In other words, memory performance was related to linear and quadratic effects of value, which in turn also affected participants’ memory estimations. However, participants appeared to have overestimated these relationships (this is also consistent with the effects sizes being higher for memory estimation than for memory performance, as reported above).

As an additional control analysis, we extended the path analysis by also including self-reported memory strength (i.e., the continuous rating of how strongly a snack was remembered, which participants gave during each cued recall trial; see Fig. 1a). Similar to memory performance, memory strength was related to linear and quadratic effects of value and partly mediated their relationship with memory estimation (Fig. S3). Importantly, the inclusion of memory strength did not diminish the direct paths from linear and quadratic value to memory estimation, suggesting that value itself (over and above memory performance and memory strength) contributed to the estimation of memory.

In light of these results, both the memory bias itself as well its dependency on the belief in value-dependent memory can be considered an (at least partially) adaptive choice strategy: If I tend to remember good things and forget bad things, then my decisions should be influenced by my knowledge about how well I remember different choice options. Given that the path analysis allows to dissociate the “true” relationships between value and memory ability (i.e., the paths from value to memory performance) from the overestimation of those relationships (i.e., the paths from value to memory estimation), we asked whether the dependency of the memory bias on memory estimation is driven by the true (linear) relationship (i.e., the blue path in Fig. 3b) or by the overestimation of this relationship (i.e., the red path in Fig. 3b). Remarkably, we found a positive correlation between the path coefficient of linear value on memory performance and the memory bias (*r*_62_ = 0.57, *P* < 0.001; left panel of Fig. 3c) but not between the path coefficient of linear value on memory estimation and the memory bias (*r*_62_ = 0.09, *P* = 0.462; right panel of Fig. 3c). This underscores the notion that participants behaved (at least partially) adaptive when they let their decisions be influenced by the fact that options were remembered or forgotten.

Notably, the relationship between memory performance and value puts the memory bias itself into question. Is the memory bias only an epiphenomenon of value-dependent memory performance (at least in the current study)? To answer this question, we calculated a *corrected memory bias* by subtracting each participant’s average standardized value of all her forgotten options from the value of the remembered option in the logistic regression that estimates the individual memory bias. The rationale of this approach is that given value-dependent memory performance, the optimal choice behavior in trials with one remembered and one forgotten option is to compare the remembered option’s value not to 0 but to the average value of forgotten options (which was indeed lower than 0; *M* = −0.11; *SD* = 0.17; *t*_63_ = −4.98, *P* < 0.001, *d* = −0.62). Expectedly, the effect size of the corrected memory bias was slightly reduced compared to the uncorrected memory bias (by about 7%), but the effect would remain significant when being tested statistically (*t*_63_ = 5.84, *P* < 0.001, *d* = 0.73; Fig. 3d). Thus, participants’ preference for remembered over forgotten options was too strong to be entirely attributable to their value-dependent memory performance.

## Discussion

In the current pre-registered study, we examined the influences of memory and beliefs about memories on value-based decisions. Specifically, to establish a mediation of memory’s impact on decision making by beliefs, we predicted to replicate the memory bias, to find that people believe to remember more desirable options more often, and to find a correlation between these two effects. All hypotheses were confirmed. In addition, our exploratory analyses revealed a quadratic relation between value and memory estimation, a pattern that is remarkably similar to the relation between value and confidence judgements^10^. Furthermore, we found a complex interplay of value, memory performance, and memory estimation: The influence of value on performance partially mediated the influence of value on memory estimation, but there were also direct influences of value on memory estimation. Our interpretation of these results is as follows: On the one hand, participants knew fairly well how often they had remembered each option in the remember-and-decide task so that the “true” relationship between value and memory induced a relationship between value and memory estimation. On the other hand, participants also believed in the influence of value on memory, so that they overestimated the true relationship. Intriguingly, not the overestimation but the true relationship was correlated with the memory bias, which characterizes the influence of memory beliefs on decisions as an adaptive process. Finally, the memory bias was not simply an artefact of the dependency of memory on value, as it persisted after correcting for this effect. In other words, even though the influence of beliefs on decisions was adaptive, the preference for better remembered options was still too strong to be considered being fully rational.

The robust dependency of an option’s memory retrieval success on its value was an unanticipated (exploratory) finding. As in our previous study, the task was designed to diminish this effect (see Methods). We speculate that differences in experimental design and sample size between our current and previous study are responsible for the diverging results: The difficulty to remember options was higher in the current study because options were always encoded only once, whereas in the previous study half of the options were encoded twice. Thus, memory performance was lower in the current study and closer to 50%, which increased the sensitivity for detecting a value-dependent (or any other) modulation. Furthermore, we tested about three times as many participants, which improved statistical power (in the previous study, there was a similar but non-significant trend). Although the value-dependency of memory performance was perhaps surprising in the context of our design, it is well in line with a wealth of previous research demonstrating that rewards can improve memory formation^11–16^. It is very likely that such value- and reward-related effects on memory extend to our every-day life. Hence, we do not consider this dependency being a confound of our main hypothesis that people’s memory beliefs mediate the memory bias. Instead, it qualifies the belief as a *correct* belief and the memory bias as a partially adaptive behavioral strategy. Allowedly, our study cannot answer the question of whether memory beliefs mediate the memory bias also in the absence of value-dependent memory performance. In our view, however, the external validity of such a scenario would be questionable in itself.

A partially adaptive memory bias on value-based decision making bears important similarities to the *Recognition Heuristic* of judgments, which was proposed within the Adaptive Toolbox research framework^17^. The Recognition Heuristic predicts that when people seek to answer statements such as “Which city has a higher population, San Diego or San Antonio?” but only recognize one of the two options, they will select the recognized option (i.e., they will judge recognized options as being larger, more important, more successful etc.). Crucially, the Adaptive Toolbox framework emphasizes the ecological rationality of the Recognition Heuristic: Using the heuristic should not be considered a fallible rule-of-thumb but an adaptive strategy, because it exploits the fact that larger and more important objects and events as well as more successful people are covered by the media more often, which in turn makes it more likely to recognize them^17,18^. Similarly, the tendency to prefer better remembered choice options when making value-based decisions appears to be (at least partially) adaptive, because–as we have argued above–better options are indeed better memorized^11–16^. Note, however, that the Recognition Heuristic has been put forward as a non-compensatory strategy, and thus predicts that people always prefer the recognized over the unknown option (but see ref.^19^). In contrast, the pattern of results related to the memory bias in our current and previous studies rather support a compensatory decision process, because participants still preferred forgotten options over very unattractive remembered options (i.e., remembered options with a standardized value of about ≥1 SD below average; see Fig. 2a). In other words, successful memory retrieval of a choice option is only one of many cues integrated during decision-making^20–23^. Again, this behavior appears to be adaptive, because it prevents the (obviously) maladaptive choice of very negative options, even though very negative options also exhibited an increased probability of being remembered in our current study (reflected in the quadratic effects of value on memory) as well as in previous research^13,14,24^.

Notably, past research investigating the influence of metacognitive beliefs about memory on judgement and decision making has revealed that retrieving information about options does not always increase evaluations and preferences^25,26^. If people experience difficulties in recalling sufficient (positive) information about an option, they may attribute this difficulty to the (presumed) fact that the option is not particularly attractive^27^. Hence, an interesting avenue for future research could be to investigate to what extent the (experienced) ease of retrieving choice options from memory modulates the memory bias on decision making, and to what extent the ease of memory retrieval is a reliable indicator of an option’s value.

Another open question for future studies is whether the belief in value-dependent memory is implicit or explicit: Although we provide clear evidence for a strong influence of value on people’s estimation of their own memory abilities, our study does not answer the question of whether people are aware of this influence (i.e., an explicit belief) or whether an option’s value also promotes its memory estimation without people knowing this (i.e., an implicit belief^28^). Similarly, it remains to be tested whether the memory bias on value-based decisions is based on an explicit belief in the inferiority of forgotten options (in the sense of a decision maker reasoning explicitly: “I do not remember this option, therefore I believe that this option cannot be particularly good, and therefore I choose another option”), or whether people are not aware that their decisions are biased by their memories.

Finally, it will be important to study the memory bias on value-based decision making with other forms of (episodic) memory. So far, our current and previous work focused on associative spatial memory, that is, participants had to link the identity of options with locations on the screen. Future studies should test whether the memory bias generalizes to other (i.e., non-spatial) forms of associative memory as well as to item memory, in particular because it has been found that these different forms of episodic memory also rely on partially distinct neural mechanisms^6,29,30^.

In conclusion, our results establish the belief in (and knowledge about) the value-dependency of successful memory retrieval as a driver of the memory bias on value-based decision making. Expectedly, the memory bias was not fully accounted for by beliefs and knowledge, and it is most likely that they are not the only cognitive mechanisms that give rise to the influence of memory on choice. Further potential impacts may issue from interactions between memory and attention as well as from the uncertainty that is necessarily entailed with the forgetting of relevant information^2^. For a comprehensive understanding of memory-based decision making, future research should investigate these additional, not mutually exclusive potential drivers of the memory bias.

## Methods

### Pre-registration

Prior to data collection, the project was pre-registered on the OSF website (https://osf.io/ze8xh/). Pre-registration comprised a description of the rationale of the study, the main hypotheses and planned statistical tests, a description of the experimental procedures (including a power analysis for sample size estimation, a list of exclusion criteria, and a statement on handling missing data), and an R-script for data analysis.

### Data Availability

The pre-registration protocol and analysis scripts are publicly available on https://osf.io/ze8xh/. Data is uploaded under the same address and also publicly available. 

### Participants

A power analysis conducted with the R-package *pwr* (function: pwr.r.test; settings: *r* = 0.3, α-level = 0.05, power = 0.80, one-sided) resulted in a desired sample size of 67 participants. Given our comparatively strict exclusion criteria (see Supplementary Information), we decided to collect data until 67 participants that passed all criteria had been tested (as outlined in the pre-registration protocol). In total, we tested 96 participants (67 female). Participants were students with an age range from 19 to 35 (*M* = 23.5, *SD* = 3.5). Data from 6 participants were not analyzed due to early termination (5) or age restrictions (1). The final sample comprised not 67 but 64 participants because 3 additional participants had to be excluded after we detected an error in the R-script that evaluated the exclusion criteria. All participants gave written informed consent, and the study was approved by the Institutional Review Board of the Department of Psychology at the University of Basel. All experiments were performed in accordance with the relevant guidelines and regulations.

### Experimental design and procedures

Similar to our previous study and related studies^2,8,31^ participants were instructed to refrain from eating for 4 hours prior to the experiment. They received course credits in addition to a maximum of two food snacks that they could gain during the experiment (see below). Additionally, participants were told to remain in the laboratory for 30 min following the experiment, during which time they were allowed to eat only the food snack(s) they won during the experiment.

After answering a demographic questionnaire, participants proceeded to the computerized tasks. Except for the estimate-your-memory task, the experimental paradigm followed the procedure described in our previous study^2^. First, participants were made familiar with a set of 48 snacks. Then, they learned to associate each snack with an intuitive abbreviation of three letters (e.g., “sni” for “Snickers”) and were asked to reproduce these abbreviations, until 100% accuracy was reached (abbreviations were required for the recall phase of the remember-and-decide task). Next, participants reported their subjective value of each snack, that is, their willingness to eat each snack at the end of the experiment on a continuous rating scale from 0 (=“not at all”) to 10 (=“very much”). After all snacks were rated once, they were presented again in a differently randomized order together with their initial ratings, and participants had the opportunity to adjust their initial ratings. Participants were incentivized to state their true preferences, as they were told that at the end of the experiment two snacks would be selected randomly, and they would receive the higher-rated snack.

Based on the value ratings, a custom-built algorithm created the trial sets for the remember-and-decide task. As in our previous study, the 6 snacks with the highest ratings were excluded, because pilot experiments of our previous study suggested that people have an exceptionally good memory for these “favorites”. Then, the algorithm created 24 for sets of 6 snacks each, ensuring that each snack occurred exactly 6 times (a requirement for the estimate-your-memory task), that each set contained a mix of high-, middle- and low-rated snacks, and that repetitions of snacks on subsequent sets were minimized (to reduce intrusion errors). Within the boundaries of these constraints, the selection of snacks was random.

The remember-and-decide task consisted of 24 blocks, each comprising 4 different phases (Fig. 1a). Each block started with the encoding phase, in which 6 snacks were presented sequentially on specific locations of the computer screen (whereas snacks varied from block to block, locations stayed the same). Each trial of this phase began with the arrangement of locations (white squares) presented for 2 s. One location was then highlighted (red square) for 1 s, followed by presenting the snack at the location for 2 s. To ensure participant’s attention, they had to indicate whether the currently presented snack was salty or sweet by pressing the keyboard button Q or P, respectively. If participants did not press a button, a message appeared for 1 s reminding them of the task. The second phase was a 2-back working memory task with 30 digits from 0 to 9, used to separate the encoding and decision phases by about 40 s and to overwrite participants’ working memory. In this task, participants had to press the ENTER key whenever the currently presented digit was the same as the digit 2 steps before. Digits were presented for 900 ms, followed by an interval of 100 ms between digits. Performance in the salty/sweet and the 2-back task was incentivized by coupling the performance in these tasks with the probability of receiving a snack at the end of the experiment. Once the 2-back task was completed, the decision phase commenced in which participants decided between 9 different snack pairs (resulting in a total of 216 decision trials). Each pair was highlighted by filling out the two respective squares with white so that participants had to recall the snacks’ identities for making decisions. They had 5 s for each decision, followed by an ITI of 2 s. If participants did not respond within 5 s, a message appeared for 1 s indicating that they were too late. Participants were instructed that they would receive the chosen snack from one randomly selected trial at the end of the experiment (in addition to the snack from the rating task) and that they would not receive this snack if they had missed in this selected trial. The last phase was a cued recall task, in which in every trial one screen location was highlighted (filled white square) and participants were required to type in the correct abbreviation (self-paced; ITI = 2 s). Participants also reported their subjective memory strength, that is, how well they thought to have remembered each recalled item on a continuous scale. Participants were informed that items could repeat across the 24 blocks of the remember-and-decide task, but that the assignment of items to locations was always new and random. In fact, all items for all participants were presented on at least two different locations (*M* = 3.99, *SD* = 0.18). Participants were trained on the remember-and-decide task for two blocks.

After completing the remember-and-decide task, participants were instructed about the estimate-your-memory task. They were informed that each snack had been presented exactly 6 times during the previous task and were asked to rate for each snack how often they thought they had successfully remembered it. Importantly, since the assignment of snacks to locations was random across the 6 presentations, participants were not asked to estimate how well they remembered each snack in a specific location, but how well they remembered it independently of the position (we provide a test of whether the snacks’ locations during the remember-and-decide task influenced participants’ memory estimates in the estimate-your-memory task in the Supplementary Information). This was done by showing each snack in the center of the screen together with 7 white dots and the digits from 0 to 6 beneath the snack (Fig. 1b). The middle dot (representing 3) was highlighted in green, and participants used the buttons Q and P to move the green dot to the left and right, respectively, when they felt they had remembered the currently presented snack less or more often. They confirmed their estimate by pressing ENTER. No time limit was imposed in this task. Following this task, participants answered questions with respect to familiarity and distinctiveness of each snack on the computer screen. They also received the food snacks they won during the experiment. Following a 30 minutes waiting period, participants received their course credit and could leave the laboratory.

Stimulus presentation and set creation were realized using MATLAB and its graphic toolbox Cogent 2000.

### Data analysis

Planned data analysis steps were outlined in the pre-registration protocol, and an R-script for data analysis was uploaded prior to data acquisition. Due to minor errors in the script, a second script had to be uploaded after data acquisition. The second script corrected the errors, highlighting all changes made. Importantly, the errors were not related to the rationale of the planned analyses.

Individual memory bias coefficients were derived as follows: Snack ratings were standardized (within participants); for decision phase trials with one remembered and one forgotten option (according to the performance in the cued recall phase), a logistic regression was calculated with the choice (remembered option chosen =1, forgotten option chosen =0) as dependent variable and the standardized rating of the remembered option as independent variable. The intercept of this logistic regression reflects the memory bias^2^. Individual coefficients for the value-dependency of memory estimation were similarly derived by a linear regression with the memory estimate of an option in the estimate-your-memory task as dependent variable and the standardized rating of the option as the independent variable. Here, a positive slope of the regression reflects a positive dependency of memory estimation on value. Coefficients for memory bias and memory estimation were checked for normality assumptions (using the Kolmogorov-Smirnoff test) before being subjected to one-sample *t*-tests against 0 (H1 and H2) and a Pearson product-moment correlation (H3).

For exploratory data analyses, we tested for a quadratic relationship between value and memory estimation. Thereto, standardized values were squared, standardized again, and added to the linear regression from H2. To calculate value-dependency of actual memory performance, a logistic regression was conducted with the memory performance in the recall phase (correct = 1, incorrect = 0) as dependent variable and the (non-squared and squared) standardized snack values as independent variables. For both regression analyses (i.e., on memory estimation and on memory performance), model comparisons between the linear-and-quadratic vs. the linear-only and vs. the quadratic-only were conducted. Thereto, the model deviances were summed over participants and the summed differences between the more general (linear-and-quadratic) model and the restricted (linear-only and quadratic-only) models were subjected to likelihood ratio tests^9^ (see also ref.^32^). In two control analyses, we checked whether the influence of value on (subjective) memory estimation remained robust when controlling for (objective) memory performance: First, we added memory performance as an independent variable when regressing memory estimation on (linear and quadratic) value (see Results). Second, we separated items according to both memory performance (i.e., how often an item was remembered) and value (i.e., whether the value of an item was below or above average), calculated the average memory estimation per performance-value pair, and then tested whether value is related to memory estimation over and above memory performance (see Fig. S1).

For the path analysis, the linear and quadratic terms of value, the actual number of correct recalls, and the estimated number of correct recalls (all standardized) were related to each other as depicted in Fig. 3b. The path coefficients of linear value on memory performance and on memory estimation were correlated with the memory bias using Pearson product-moment correlations (after checking for normality assumptions). In a second path analysis, we also included self-reported memory strength as depicted in Fig. S3. The “corrected” memory bias was derived by first calculating the average value of forgotten options in each participant, and then subtracting this value from the standardized rating of the remembered option in the logistic regression.

For the hypothesis-driven analyses, one-sided *P*-values are reported. For exploratory analyses, two-sided *P*-values are reported (but inferential statistical tests for exploratory analyses should be treated with caution). Data analyses were conducted in R and in MATLAB and results were compared as a robustness check. The path analysis was conducted in R only, using the R-package *lavaan*.

## Electronic supplementary material

Supplementary Information

## References

[1] Weber, E. U. & Johnson, E. J. Constructing preferences from memory. In *The Construction of Preference* (eds Lichtenstein, S. & Slovic, P.) 397–410 (Cambridge University Press, 2006).

[2] Gluth S, Sommer T, Rieskamp J, Büchel C (2015). Effective connectivity between hippocampus and ventromedial prefrontal cortex controls preferential choices from memory. Neuron.

[3] Shadlen MN, Shohamy D (2016). Decision making and sequential sampling from memory. Neuron.

[4] Murty, V. P., FeldmanHall, O., Hunter, L. E., Phelps, E. A. & Davachi, L. Episodic memories predict adaptive value-based decision-making. *J. Exp. Psychol. Gen*. **145,** 548–558 (2016). 10.1037/xge0000158 PMC4833575 26999046

[5] Wimmer GE, Büchel C (2016). Reactivation of reward-related patterns from single past episodes supports memory-based decision making. J. Neurosci. Off. J. Soc. Neurosci..

[6] Weilbaecher RA, Gluth S (2017). The interplay of hippocampus and ventromedial prefrontal cortex in memory-based decision making. Brain Sci..

[7] Eichenbaum H (2017). Prefrontal–hippocampal interactions in episodic memory. Nat. Rev. Neurosci..

[8] Levy DJ, Glimcher PW (2012). The root of all value: A neural common currency for choice. Curr. Opin. Neurobiol..

[9] Farrell, S. & Lewandowsky, S. *Computational Modeling of Cognition and Behavior*. (Cambridge University Press, 2018).

[10] Lebreton M, Abitbol R, Daunizeau J, Pessiglione M (2015). Automatic integration of confidence in the brain valuation signal. Nat. Neurosci..

[11] Adcock RA, Thangavel A, Whitfield-Gabrieli S, Knutson B, Gabrieli JDE (2006). Reward-motivated learning: mesolimbic activation precedes memory formation. Neuron.

[12] Wittmann BC (2005). Reward-related fMRI activation of dopaminergic midbrain is associated with enhanced hippocampus-dependent long-term memory formation. Neuron.

[13] LaBar KS, Cabeza R (2006). Cognitive neuroscience of emotional memory. Nat. Rev. Neurosci..

[14] Shohamy D, Adcock RA (2010). Dopamine and adaptive memory. Trends Cogn. Sci..

[15] Cohen MS, Rissman J, Suthana NA, Castel AD, Knowlton BJ (2014). Value-based modulation of memory encoding involves strategic engagement of fronto-temporal semantic processing regions. Cogn. Affect. Behav. Neurosci..

[16] Cohen MS, Rissman J, Hovhannisyan M, Castel AD, Knowlton B (2017). J. Free Recall Test Experience Potentiates Strategy-Driven Effects of Value on Memory. J. Exp. Psychol. Learn. Mem. Cogn..

[17] Goldstein DG, Gigerenzer G (2002). Models of ecological rationality: The recognition heuristic. Psychol. Rev..

[18] Todd P, Gigerenzer G (2007). Environments that make us smart..

[19] Pachur T (2011). The limited value of precise tests of the recognition heuristic. Judgm. Decis. Mak..

[20] Glöckner A, Bröder A (2014). Cognitive integration of recognition information and additional cues in memory-based decisions. Judgm. Decis. Mak..

[21] Pohl RF (2006). Empirical tests of the recognition heuristic. J. Behav. Decis. Mak..

[22] Richter T, Späth P (2006). Recognition is used as one cue among others in judgment and decision making. J. Exp. Psychol. Learn. Mem. Cogn..

[23] Heck, D. W. & Erdfelder, E. Linking process and measurement models of recognition-based decisions. *Psychol. Rev*. **124**, 442–471 (2017). 10.1037/rev0000063 28368144

[24] Hamann S (2001). Cognitive and neural mechanisms of emotional memory. Trends Cogn. Sci..

[25] Schwarz N (1991). Ease of retrieval as information: Another look at the availability heuristic. J. Pers. Soc. Psychol..

[26] Wänke M, Bohner G, Jurkowitsch A (1997). There are many reasons to drive a BMW: Does imagined ease of argument generation influence attitudes?. J. Consum. Res..

[27] Schwarz N (2004). Metacognitive experiences in consumer judgment and decision making. J. Consum. Psychol..

[28] Greenwald AG, McGhee DE, Schwartz JL (1998). Measuring individual differences in implicit cognition: the implicit association test. J. Pers. Soc. Psychol..

[29] Davachi L (2006). Item, context and relational episodic encoding in humans. Curr. Opin. Neurobiol..

[30] Eichenbaum H, Yonelinas AP, Ranganath C (2007). The medial temporal lobe and recognition memory. Annu. Rev. Neurosci..

[31] Krajbich I, Armel C, Rangel A (2010). Visual fixations and the computation and comparison of value in simple choice. Nat. Neurosci..

[32] Boll S, Gamer M, Gluth S, Finsterbusch J, Büchel C (2013). Separate amygdala subregions signal surprise and predictiveness during associative fear learning in humans. Eur. J. Neurosci..

