# Supplementary material for: Memory Beliefs Drive the Memory Bias on Value-based Decisions
Source: Sci Rep. 2018 Jul 12;8:10592. doi: 10.1038/s41598-018-28728-9 (PMC6043538; doi:10.1038/s41598-018-28728-9)
Supplement: Supplementary file 1 — Supplementary Information [file 41598_2018_28728_MOESM1_ESM.docx]

**Memory Beliefs Drive the Memory Bias on Value-based Decisions**

**Tehilla Mechera-Ostrovsky & Sebastian Gluth**

SUPPLEMENTARY INFORMATION

**
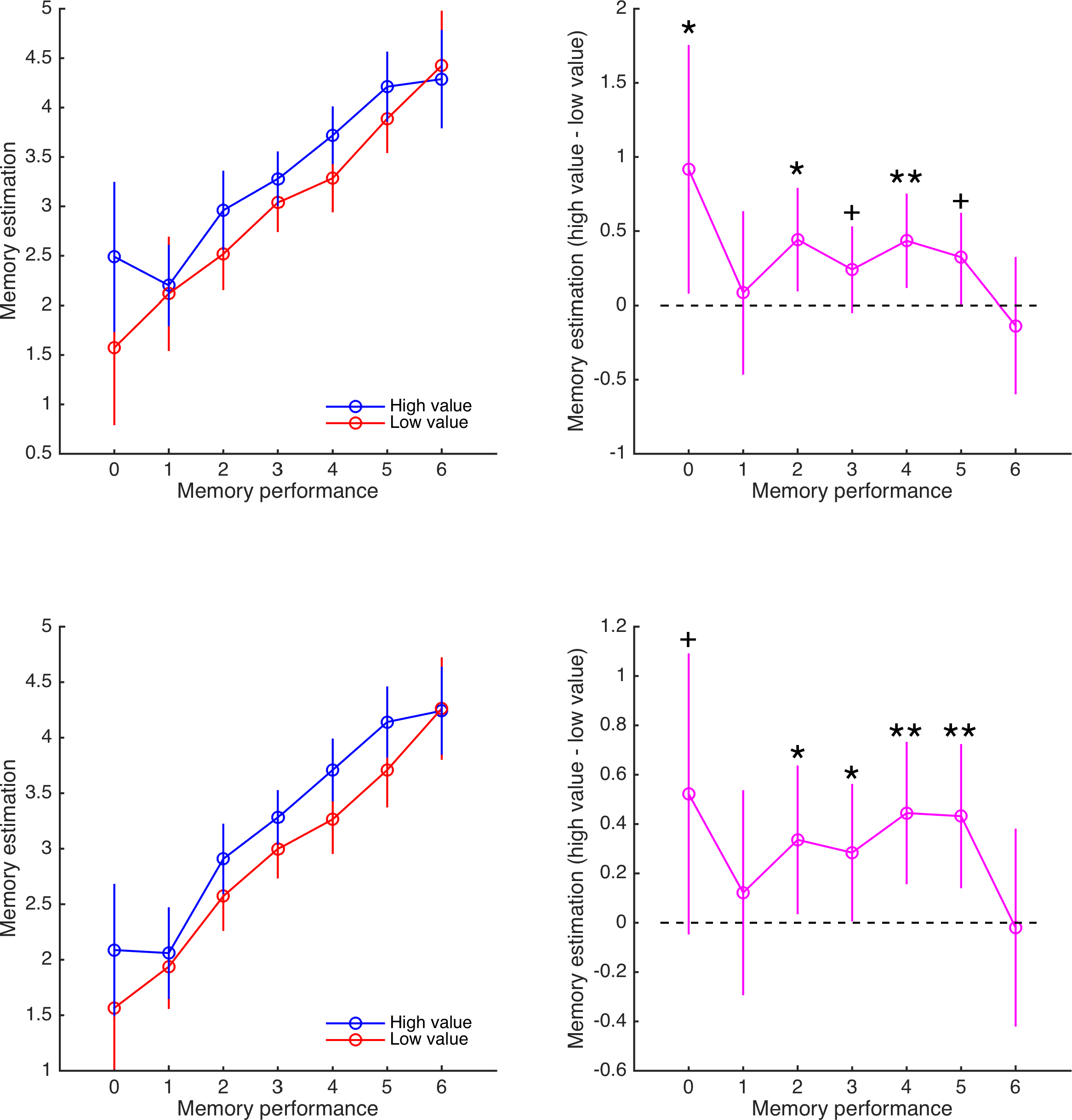
**

**Figure S1** Disambiguating between contributions of value and memory performance to memory estimation. Upper panels show results for the restricted sample, lower panels show the results for the full sample. The left panel depicts the estimated memory performance as a function of memory performance (i.e., how often an item was remembered correctly), separately for high-value items (i.e., standardized value > 0; blue line) and low-value items (i.e., standardized value < 0; red line). The right panel shows the high-low value difference in memory estimation to better illustrate statistical significance. For six out of seven memory-performance levels, high-value items were estimated to be remembered better than low-value items. Collapsing over all memory-performance levels yielded strong evidence that memory estimation was driven by value (restricted sample: *t*_63_ = 4.01, *P* < .001, *d* = 0.50; full sample: *t*_89_ = 4.61, *P* < .001, *d* = 0.49) – over and above the effect of memory performance. Error bars represent 95% CIs. ^+^*P* < .1, **P* < .05, ***P* < .01


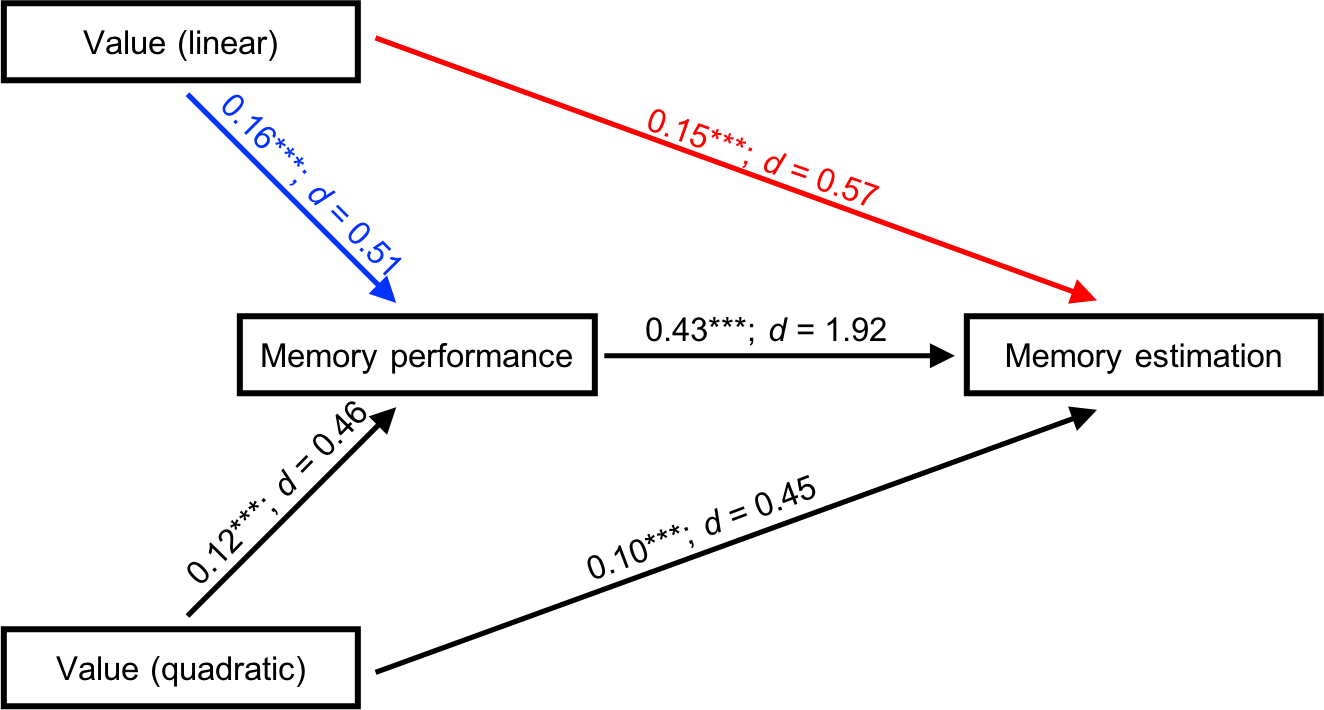


**Figure S2** Path analysis results for the full sample. For the full sample, the correlation between the (blue) path from value (linear) to memory performance with the memory bias was *r*_88_ = .52, *P* < .001, and the correlation between the (red) path from value (linear) to memory estimation with the memory bias was *r*_88_ = .17, *P* = .100. ****P* < .001


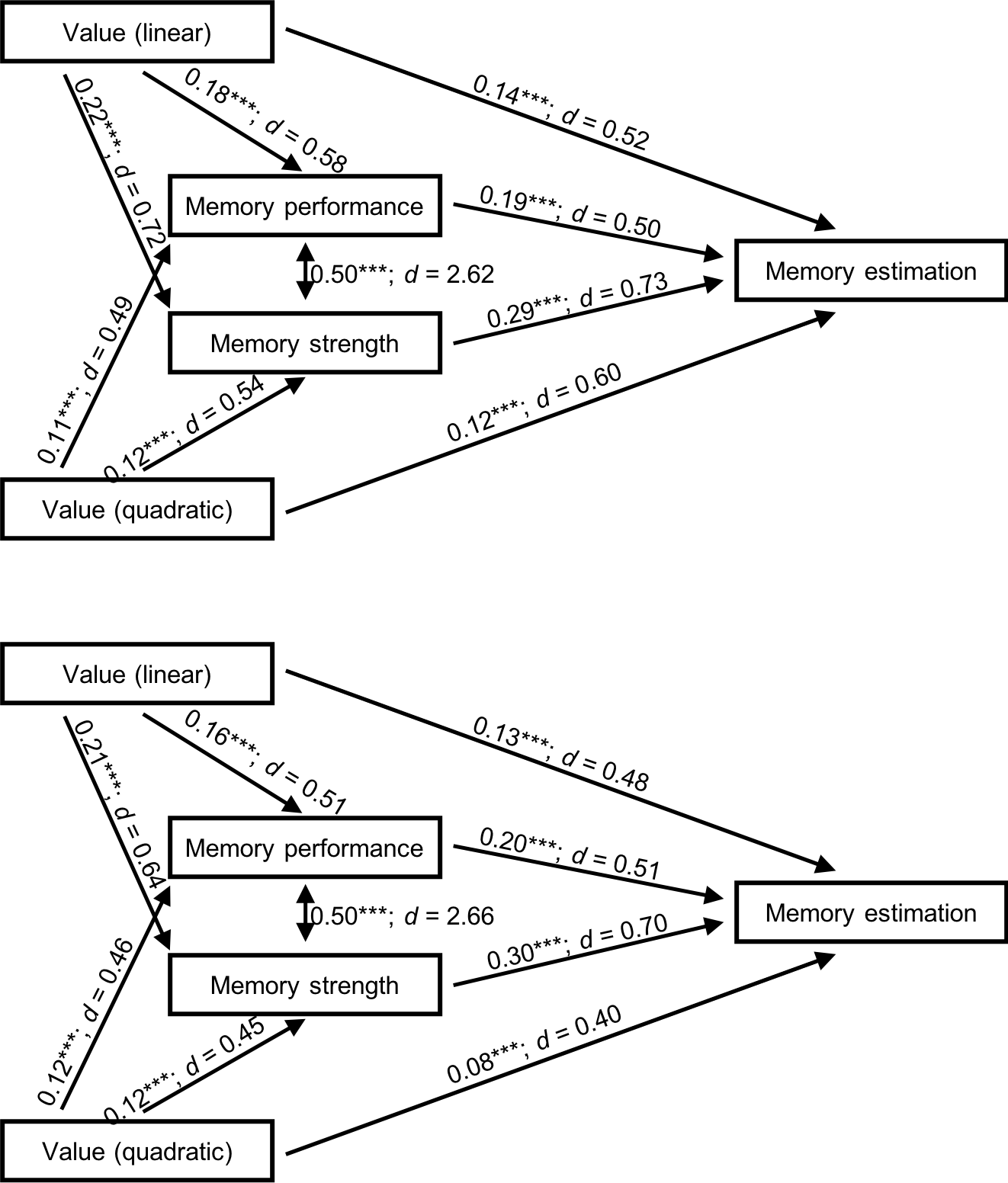


**Figure S3** Extended path analyses for the restricted (upper panel) and full (lower panel) samples. For the extended path analyses, the (subjective and continuous) memory strength was included next to the (objective and binary) memory performance. Expectedly, memory strength and memory performance were strongly related to each other (see the coefficient of the bi-directional arrow between them). However, both variables contributed independently to predicting memory estimation. Most importantly, the direct paths from value to memory estimation remained significant, suggesting that the influence of value on memory estimation cannot be solely attributed to memory performance or memory strength. ****P* < .001

**Supplementary Methods**

**Stimulus material.** Prior to the main study, we conducted a pilot study to determine an adequate set of diverse, likeable and well-known snacks with easily memorable abbreviations. We tested 60 snacks that are available at typical Swiss supermarkets to fit 6 categories (salty snacks, soft candies, hard candies, healthy bars, chocolate bars, nuts). We asked a different pool of 21 participants (15 female) to rate each snack for its familiarity, distinctiveness as well as how much they liked each snack and how well it represented the matching category. Participants in this pilot study were undergraduate with an age range from 18 to 29 (*M* = 21.8, *SD* = 2.98). Based on these evaluations, we selected 48 food snacks for our main study (i.e., particularly unattractive and/or unknown snacks were excluded).

**Exclusion criteria.** Our experimental procedures, especially the remember-and-decide task, are comparatively effortful and demand full attention. This fact could lead participants to partially give up on demanding parts of the tasks (e.g., they could ignore the 2-back task to facilitate remembering the options for the decision phase). Moreover, some of our analyses require task performances to be within certain ranges (e.g., the memory bias coefficient cannot be estimated, if a participant remembers (almost) everything or (almost) nothing, as there would not be enough trials with one remembered and one forgotten option). Accordingly, we defined a list of exclusion criteria that are described in details in our pre-registration protocol. These restrictions were added to the personal/demographic restrictions (e.g., age, mental disorders). The listed exclusion criteria comprised too extreme ratings of snacks (>40% of minimum or maximum ratings), too many missed responses in the salty/sweet task (>40%), too low performance in the 2-back task (<70% accuracy), too fast decisions in the decision phase (>30% of trials with <250 ms response time), too low / too high performance in the recognition phase (<30% / >85% accuracy), and too extreme memory estimates in the estimate-your-memory task (>50% of estimates either 0 or 6). Furthermore, we excluded participants, if their regression coefficients for the memory bias or for the value-dependency of memory estimation were >3 SD lower or higher than the group’s average (to prevent excessive influences of outliers on the correlation).

From the 26 excluded participants, 9 participants were excluded because of too extreme ratings of snacks, 8 because of too low 2-back task performance, 4 because of too low recognition performance, 2 because of too extreme ratings of snacks and too low recognition performance, 1 because of too extreme ratings of snacks and too low 2-back task performance, 1 because of too low recognition performance and too low 2-back task performance, and 1 because of too low recognition performance and too extreme memory estimates. As shown in Table 1, including these participants into the analyses does not change any results qualitatively.

**Supplementary Results**

**Control analysis for screen locations.** As an additional robustness check of hypothesis H2 (i.e., that people estimate their memory to be higher for more valuable items), we checked whether the snacks’ locations on the screen during the remember-and-decide task had an influence on participants’ memory estimates in the estimate-your-memory task. Thereto, we calculated three scores for each snack: i.) the “average left-right position” of each snack (with higher values indicating that a snack was shown more often on locations to the right of the screen), ii.) the “average up-down position” of each snack (with higher values indicating that a snack was shown more often in the upper row of locations), and iii.) a “randomization of position” index for each snack (i.e., on how many different positions a snack was presented). These three scores were added to the analysis that regressed memory estimation on (linear and quadratic) value, and that also controlled for memory performance. Neither the “average left-right position” (restricted sample: *t*_63_ = 0.44, *P* = .658, *d* = 0.06; full sample: *t*_89_ = -0.71, *P* = .479, *d* = -0.07), nor the “average up-down position” (restricted sample: *t*_63_ = -1.24, *P* = .221, *d* = -0.15; full sample: *t*_89_ = -0.36, *P* = .723, *d* = -0.04), nor the “randomization of position” (restricted sample: *t*_63_ = 0.34, *P* = .722, *d* = 0.04; full sample: *t*_89_ = 0.27, *P* = .788, *d* = 0.03) predicted memory estimates significantly. Moreover, adding these variables did not diminish the linear effect of value on memory estimation (restricted sample: *t*_63_ = 3.94, *P* < .001, *d* = 0.49; full sample: *t*_89_ = 4.68, *P* < .001, *d* = 0.49). Taken together, the influence of value on memory estimation (i.e., our hypothesis H2) remained robust even after controlling for both memory performance and screen locations of the items.
